# Evolution of Cognitive Impairments in Treatment-Resistant Depression: Results from the Longitudinal French Centers of Expertise for Treatment-Resistant Depression (FACE-DR) Cohort

**DOI:** 10.3390/brainsci13071120

**Published:** 2023-07-24

**Authors:** Alexis Vancappel, Yecodji Dansou, Ophelia Godin, Emmanuel Haffen, Antoine Yrondi, Florian Stephan, Raphaelle Marie Richieri, Fanny Molière, Jérôme Holtzmann, Mathilde Horn, Etienne Allauze, Jean Baptiste Genty, Alex Bouvard, Jean-Michel Dorey, Vincent Hennion, Vincent Camus, Guillaume Fond, Barbara Peran, Michel Walter, Loic Anguill, Charlotte Scotto D’apolina, Estelle Vilà, Benjamin Fredembach, Jean Petrucci, Romain Rey, Anne Sophie Nguon, Bruno Etain, Mathilde Carminati, Philippe Courtet, Guillaume Vaiva, Pierre Michel Llorca, Marion Leboyer, Bruno Aouizerate, Djamila Bennabi, Wissam El Hage

**Affiliations:** 1 Fondation FondaMental, 94000 Créteil, France; yecodji.dansou@etu.univ-nantes.fr (Y.D.); jeanbaptiste.genty@aphp.fr (J.B.G.); jean-michel.dorey@ch-le-vinatier.fr (J.-M.D.); michel.walter@chu-brest.fr (M.W.); benjaminfredembach@chu-grenoble.fr (B.F.); romain.rey@ch-le-vinatier.fr (R.R.);; 2 CHRU de Tours, UMR 1253, iBrain, Université de Tours, Inserm, 37000 Tours, France; 3 EE 1901 Qualipsy, Université de Tours, 37000 Tours, France; 4 INSERM U955, Équipe de Psychiatrie Translationnelle, Université Paris-Est Créteil, DHU Pe-PSY, Pôle de Psychiatrie des Hôpitaux Universitaires H Mondor, 94000 Créteil, France; 5 Department of Clinical Psychiatry, CIC-1431 INSERM, CHU de Besançon, UR481 Neurosciences and Cognition, University of Franche-Comté, 25000 Besançon, France; 6 Service de Psychiatrie et de Psychologie Médicale de l’adulte, CHU de Toulouse, Hôpital Purpan, Université Paul Sabatier Toulouse 3, 31062 Toulouse, France; 7 Service Hospitalo-Universitaire de Psychiatrie Générale et de Réhabilitation Psycho Sociale, 29200 Brest, France; 8 Equipe Imothep, Institut Fresnel, UMR 7249, Aix-Marseille Université, CNRS, Ecole Centrale Marseille, 13284 Marseille, France; 9 Department of Emergency Psychiatry and Post Acute Care, Hôpital Lapeyronie, CHU Montpellier, 34000 Montpellier, France; 10 Service Hospitalo-Universitaire de Psychiatrie, CHU Grenoble Alpes, University Grenoble Alpes, 38400 Grenoble, France; 11 Centre de Référence Régional des Pathologies Anxieuses et de la Dépression, Centre Expert Dépression Résistante, Pôle de Psychiatrie Générale et Universitaire, CH Charles Perrens, 33076 Bordeaux, France; 12 Laboratoire Nutrition et Neurobiologie Intégrée (UMR INRAE 1286), Université de Bordeaux, 33076 Bordeaux, France; 13 INSERM UMR-S 1144 Optimisation Thérapeutique en Neurospsychopharmacologie, Département de Psychiatrie et de Médecine Addictologique, OTeN, Hôpitaux Lariboisière-Fernand Widal, GHU APHP Nord_Université Paris Cité, 75006 Paris, France; 14 Clinical Research Unit, Academic Hospitals of Marseille (APHM), School of Medicine–La Timone Medical Campus, EA 3279, Department of Epidemiology and Health Economics, Aix-Marseille University, 13284 Marseille, France; 15 Inserm-U1172–LilNCog–Lille Neuroscience & Cognition, Centre National de Ressources & Résilience pour les Psychotraumatismes (Cn2r Lille Paris), Université de Lille (CHU Lille), 59000 Lille, France

**Keywords:** treatment-resistant depression, cognitive impairments, neuropsychology, memory, executive function, processing speed

## Abstract

Previous studies set out profound cognitive impairments in subjects with treatment-resistant depression (TRD). However, little is known about the course of such alterations depending on levels of improvement in those patients followed longitudinally. The main objective of this study was to describe the course of cognitive impairments in responder versus non-responder TRD patients at one-year follow-up. The second aim was to evaluate the predictive aspect of cognitive impairments to treatment resistance in patients suffering from TRD. We included 131 patients from a longitudinal cohort (FACE-DR) of the French Network of Expert TRD Centers. They undertook comprehensive sociodemographic, clinical, global functioning, and neuropsychological testing (TMT, Baddeley task, verbal fluencies, WAIS-4 subtests, D2 and RLRI-16) at baseline (V0) and one-year follow-up (V1). Most patients (*n* = 83; 63.36%) did not respond (47 women, 49.47 ± 12.64 years old), while one-third of patients responded (*n* = 48, 30 women, 54.06 ± 12.03 years old). We compared the cognitive performances of participants to average theoretical performances in the general population. In addition, we compared the cognitive performances of patients between V1 and V0 and responder versus non-responder patients at V1. We observed cognitive impairments during the episode and after a therapeutic response. Overall, each of them tended to show an increase in their cognitive scores. Improvement was more prominent in responders at V1 compared to their non-responder counterparts. They experienced a more marked improvement in code, digit span, arithmetic, similarities, and D2 tasks. Patients suffering from TRD have significant cognitive impairments that persist but alleviate after therapeutic response. Cognitive remediation should be proposed after therapeutic response to improve efficiency and increase the daily functioning.

## 1. Introduction 

Major Depressive Disorder (MDD) [1] is a serious public health issue [2]. Indeed, its lifetime prevalence is high among the French population (15–20%) [3,4], and one in every three patients suffering from MDD has to stop working at some point [5]. Indeed, MDD causes functional impairment that seems to be mediated by cognitive dysfunction [6]. Consistently, meta-analyses have showed the presence of cognitive disturbances among depressed patients, affecting multiple cognitive processes such as verbal memory, processing speed, and executive function [7,8,9]. Moreover, some results indicate a positive association between depressive symptoms and cognitive deficits [10]. Other works also suggest that cognitive impairments are associated with a poorer response to antidepressant treatment or with more residual symptoms [11,12,13,14]. Finally, such impairments seem to be present during the first episode [15] and remain after remission [8,16,17,18]. As we said earlier, this result is important as cognitive impairments are associated with functional impairment such as social and occupational alterations [19]. In line with this, some studies have highlighted that cognitive impairments are the mediator between functional alterations and MDD [20,21]. More specifically, one of them showed that the relationship between MDD and functional impairment was fully mediated by executive dysfunction [22].

Even if the presence of cognitive impairments seems clear in MDD, there is a lack of studies performed among patients suffering from treatment-resistant depression (TRD), defined as a non-response to at least two different classes of antidepressant treatments at appropriate dosage and time treatment [23]. To the best of our knowledge, only three studies have been performed. The first one compared 53 patients suffering from a first episode of MDD and 53 patients suffering from TRD. The results found poorer performance in executive tasks (TMT-B, Wisconsin Card Sorting Task, and Towers of London) by patients suffering from TRD as compared to those experiencing a first episode [24]. A second study set out that cognitive deficit is among the significant predictive factors of treatment resistance in MDD [12]. This study compared 125 non-TRD subjects to 104 patients suffering from TRD. They found that TRD was associated with worse performances concerning verbal memory, processing speed, and executive function. Finally, a large cohort study found that TRD patients showed serious cognitive impairments that are associated with poorer daily functioning [25]. Taken together, all these findings suggest that cognitive impairments should be a target and that cognitive remediation trials could be successfully proposed for managing such patients and therefore improving daily functioning, e.g., [26]. However, it is still unclear when such interventions should be delivered. Indeed, if patients suffering from MDD partly recover their cognitive abilities, it would be irrelevant to propose cognitive remediation during the episode. Such interventions would be more appropriate after therapeutic response or a remitted state. Moreover, no study has explored if cognitive impairments could be predictive for a therapeutic nonresponse within patients suffering from TRD.

Therefore, the main objective of this study was to describe the course of cognitive impairments in responder versus non-responder TRD patients at one-year follow-up. The second aim was to evaluate the predictive aspect of cognitive impairments to treatment resistance in patients suffering from TRD.

## 2. Method

### 2.1. Study Design

Participants were recruited from 13 Centers of Expertise for Treatment-Resistant Depression (FACE-DR) that all followed the same standardized clinical assessments [27]. To be considered a FACE-DR center, each investigator site had to employ at least a secretary involved in monitoring patients’ appointments and one psychiatrist along with one neuropsychologist trained in the standardized, comprehensive, and multidimensional assessment of FACE-DR that was performed every year for four consecutive years. Psychiatrists undertook at least yearly clinical evaluations of patients’ videos to ensure concordance among raters. Due to the important rate of dropout, we proposed to consider the data primarily related to depression severity, neuropsychological impairment, and daily functional disability and collected during the first two visits at the one-year interval. We used the following outcomes of the battery: neuropsychological functioning, daily functioning, and depression. 

### 2.2. Settings

The Centers of Expertise for Treatment-Resistant Depression (FACE-DR) were widely distributed over 13 French cities, including Paris/Créteil, Paris Fernand-Widal, Besançon, Bordeaux, Brest, Clermont-Ferrand, Grenoble, Marseille, Montpellier, Lille, Lyon, Toulouse, and Tours. To be eligible for the study, patients had to be adults and suffer from treatment-resistant depression. 

### 2.3. Participants 

Depressed patients were recruited and assessed individually in ambulatory settings. We recruited 131 depressed (83 women) patients. As described elsewhere [27], they met DSM-IV criteria for major depression. They experienced depressive symptom intensities corresponding to scores above 19 on the MADRS. They were clinically unresponsive to two successive and adequate sequences of antidepressants from two different pharmacological classes corresponding to at least stage II of the staging criteria proposed by Thase for defining TRD [28]. The exclusion criteria were suffering from bipolar disorders, psychotic disorders, OCD, eating disorders (with BMI  <  15), somatoform disorders, and mood disorders related to substance abuse or misuse. From the general cohort (*n* = 397), we included only patients who fully completed the first (inclusion visit, V0) and the second visits (1 year later, V1) (*n* = 183). From the latter, we removed patients with a history of electro-convulsive therapy (ECT) during the last 6 months, sensory disorder, dyslexia, dysorthographia, dyscalculia, dysphasia, dyspraxia, language delay, epilepsy, meningitis, or multiple sclerosis. We also excluded patients who did not undergo depression and/or neuropsychological evaluations in one of two visits. In the end, 131 patients including 48 men and 83 women with the mean age of 51.22 (12.44) and the mean education level of 13.37 (2.91) (12 corresponding to high school diploma) meeting all the criteria were considered for this study. The neuropsychological evaluation took two hours, and the order of the tests was fixed. Some patients were not able or did not accept to perform all tests, explaining the missing data. At V1, we distinguished responder and non-responder patients. Patients were considered as responders if they showed a reduction of at least 50% of their Montgomery–Åsberg Depression Rating Scale (MADRS) score at V1. The characteristics of the overall population are presented in Table 1 and Table 2.

### 2.4. Variables

#### 2.4.1. Clinical Assessment

At baseline, a trained psychiatrist interviewed the participants using the DSM-IV Mini International Neuropsychiatric Interview (MINI). He also collected information about the patient’s education, marital status, onset and course of MDD, clinical features, and psychiatric comorbidities. Education level was determined as the number of school years from the first year of primary school. Twelve years corresponds to a high school diploma. We then included different standardized measures. Depression severity was rated and coupled with the evaluation of executive functions and processing speed, as previous studies already set out serious impairments among these cognitive processes in depressed subjects [8,25]. The impact of depression and related cognitive alterations on daily functioning was also assessed 6. Then, we used the following standardized measures:

- The Montgomery–Åsberg Depression Rating Scale—MADRS [29].

This is a 10-item semi-structured interview that allows for the measurement of depressive symptoms. Clinicians had to rate the different symptoms based on verbal or non-verbal information. Each item scored between 0 and 6. A greater score indicates more severe depressive symptoms. The French version of the MADRS presents a good internal consistency (Cronbach’s α from 0.85 to 0.94) [30]. The MADRS also presents a good sensitivity and specificity, 66 and 60%, respectively [31].

- The Functional Assessment Short Test—FAST [32].

This is a 24-item semi-structured interview assessing daily functioning across different areas (autonomy, occupational functioning, cognitive functioning, financial problems, interpersonal problems, and hobbies). A greater score indicates more important difficulties. The French version of the FAST demonstrated a good internal consistency (Cronbach’s α = 0.97) and a good validity [33].

#### 2.4.2. Neuropsychological Testing

- The RL/RI-16 task.

This task evaluates verbal episodic memory. Patients had to learn 16 words associated with 16 cues [34]. Thereafter, they had to recall as many words as possible for two minutes. After this time, cues were given to the patient if necessary. Three free and cued recalls were performed immediately after learning with a distractive task between each recall. A recognition task was also performed. Patients were asked to recognize the words learned among distractors. Finally, a delayed recall was conducted 20 min after the third recall.

- The D2 task.

This task assesses focused and sustained attention [35]. Patients had to cross d with two lines, among distractors.

- The trail making test (TMT).

This test measures visual scanning and flexibility [36]. In the first condition (TMT-A), patients were invited to connect numbers in ascending order. In the second condition (TMT-B), they were asked to connect alternating numbers and letters in an assembling or alphabetic order.

- The coding subtest of the WAIS-IV.

This test measures processing speed [37]. The patients were asked to copy as many symbols as possible, depending on a discriminative stimulus over two minutes.

- The symbol subtest of the WAIS-IV.

This test measures processing speed [37]. The patients were required to search and cross symbols among distractors.

- The arithmetic subtest of the WAIS-IV.

This test assesses working memory [37]. The patients had to orally solve mathematical problems.

- The digit span subtest of the WAIS-IV.

This test evaluates working memory [37]. The patients had to recall digit sequences in front order, back order, and ascending order.

- The similarities subtest of the WAIS-IV.

This test assesses verbal knowledge and abstraction [37]. The patients had to explain the similarities between two concepts.

- The verbal fluencies test.

This test evaluates lexical access and flexibility [38]. In the first condition, patients had to provide as many words as possible belonging to a given semantic category (semantic fluencies). In a second condition, participants had to deliver as many words beginning with a letter as possible (phonological fluencies).

- The double Baddeley task.

In this task, participants had to cross a line and then perform a digit span task [38]. They performed these tasks separately and then undertook both tasks simultaneously. This allows us to calculate a Mu score comparing the performance of patients while doing two tasks separately and simultaneously. These measure coordination abilities recruiting the central executive system in the working memory model [39].

### 2.5. Statistical Analysis

We first performed the power analysis. We used Gpower to do so. By convention, we fixed the alpha value at 0.05 and the beta value at 0.10. Based on a previous meta-analysis performed on cognitive deficits among depressed patients [8], we fixed the effect size as d = 0.38. This corresponds to the mean size of the smallest effect found on cognition. In this situation, the software proposes a sample size of 61 participants to identify such an effect.

We used normative data to transform raw scores from the neuropsychological tasks into normalized scores; 0 represents the mean and 1 represents the standard deviation. Then, the normality of the distribution was first assessed through graphical representations and showed an acceptable fit to continue with parametric tests. After that, we performed every further analysis using the normalized scores. First, we compared the mean performances of the patients to the mean 0 to assess the cognitive impairments. Only D2 scores were calculated in percentiles and involved a comparison to a different mean (m0 = 50). Then, we used paired *t* test comparisons to assess the evolution of the patients’ performances from V0 to V1. We calculated the delta of the different scores (Δ = V1scores − V0 score) and evaluated the correlations between the MADRS delta and the neuropsychological standardized scores delta. Finally, we used *t* tests to compare the performances of responder (*N* = 48) and non-responder (*N* = 83) patients at V1. Statistical analysis was performed using SPSS, 23rd version.

## 3. Results

### 3.1. Descriptive Analyses

At V1, among the 131 participants, two-thirds did not respond (*n* = 83, 47 women), while one-third responded (*n* = 48; 30 women). Descriptive data are displayed in Table 1 and Table 2.

### 3.2. Comparison to the Norm

For the complete study sample, at baseline, TRD patients performed worse than the norm of the general population expected for all tasks, except for in the TMT-A, Baddeley task, phonologic fluencies, and similarities test. At M12, the cognitive performances of participants did not significantly differ from the average theoretical performance of the general population for the symbol task, TMT-B task, arithmetic task, and draw 1 task.

At M0, the group of responder patients performed worse than the norm of the general population in all tasks, except for the TMT-A and -B, similarities, and draw 2 tasks. However, at M12, the difference from the norm was not significant for the symbol task, digit span, arithmetic task, free recall 1, and draw 1.

Finally, when we compared the D2 percentile scores to the mean 50, we found that the patients’ scores were always under the norm except at V0 and V1 and for both responder and non-responder patients (*t* between −7.73 and −2.18 and *p* < 0.05). The details of the results are presented in Table 3.

### 3.3. Comparison between V0 and V1

Among non-responder patients, we found a significant improvement in cognitive performances from V0 to V1 in multiple scores: MADRS, symbol task, TMT-B, free recall 1, and draw 1. We found even more evolution among responder patients, who improved in the MADRS, code task, symbol task, digit span, arithmetic task, similarities, D2, free recall 1 and 3, and draw 2. The details of the results are presented in Table 4.

### 3.4. Correlation between Δ-Depression and Δ-Neuropsychological Tasks

We only found a significant correlation between Δ-MADRS and the Δ-D2 (*r* = −0.26; *p* = 0.037; *N* = 62) and the Δ-digit span (*r* = −0.23; *p* = 0.044; *n* = 74).

### 3.5. Comparison between Responder and Non-Responder Patients

Comparing responder and non-responder patients, we found that responder patients performed worse at V0 in verbal fluencies. In contrast, they showed a greater score for the MADRS score and in the arithmetic task. The details of the results are presented in Table 5.

## 4. Discussion

The aim of this study was to characterize the course of cognitive impairments among patients suffering from TRD over a one-year follow-up. Consistent with previous results, we found the existence of cognitive impairments among both non-responder [7,8,9] and responder TRD patients [8,16,17,18]. The cognitive impairments were preferentially observed for speed processing, executive functions, and episodic memory. Cognitive impairment was less marked at one year compared to baseline, thereby leading us to consider that there may be an alleviation of cognitive impairments over time in TRD patients. This improvement was found for all cognitive areas and was more important for responder than non-responder patients at M12. This observation strengthens the idea of a causal relationship between depressive symptoms and cognitive impairments. Overall, the multiple forms of cognitive impairments support the global-diffuse hypothesis suggesting that cognitive deficit in depression is rather due to an overall decline in attention and not to specific alterations of distinct functions [40,41]. Moreover, earlier studies have supported that the cognitive disturbances were associated with a reduced connectivity of the fronto-parietal control system in depressed subjects. This may explain a more important engagement in negative self-referential thoughts than in the external environment [42].

This study also aimed to evaluate the potential prediction of neurocognitive performance on therapeutic response among patients suffering from TRD. Surprisingly, we found that responder patients perform worse in verbal fluency at baseline than non-responder patients. This is incongruent with previous results supporting the notion that cognitive impairments are associated with a poorer response to antidepressants [11,12,13,14]. This may suggest that cognitive impairments are not a significant predictor of therapeutic response among patients suffering from TRD. Another explanation could be that cognitive impairments are able to predict clinical outcomes after the first line of antidepressants but not the response to subsequent therapies.

These findings are particularly relevant for the further development of personalized and innovative treatment strategies targeting cognitive deficits. Indeed, it seems that depression contributes to significantly altered cognitive functioning. Therefore, it may be more relevant to evaluate the patient’s cognitive impairments after a therapeutic response. The main focus during the episode should be the treatment of the depressive episode, as cognitive impairments tend to reduce with symptom reduction. The treatment of cognitive impairments may follow. At this point, cognitive remediation should be proposed to the patients to increase their daily functioning.

Even if we know that patients suffering from MDD present cognitive impairments during the episode and after a therapeutic response, it remains unclear if such impairments are already present before the episode. Longitudinal studies could focus on such questions to identify the potential causal role of cognitive impairments in the development of MDD.

## 5. Limitations

The main limit of our study was the comparison of the patients’ performances to theoretical performances. Indeed, a more adapted methodological approach would have been the classical use of a control group. A second limit is a reduced statistical power among this sample. Furthermore, we only performed a one-year follow-up, while depression is a chronic disease. Further studies using long-term follow-up should focus on the evolution of cognitive impairments. Finally, we did not control confounding variables in order not to reduce the statistical power. In this context, the impact of concomitant medical treatments such as benzodiazepines/hypnotics on cognitive functions should further be taken into account in larger cohort studies on TRD.

## 6. Conclusions

Patients suffering from TRD have cognitive impairments affecting especially processing speed, executive functions, and verbal episodic memory during and after the episode. However, cognitive functioning tends to improve in relation to the alleviation of depressive symptoms. This suggests that cognitive impairments should be targeted in the second stage of therapeutic intervention.

## Figures and Tables

**Table 1:** (**a**) Descriptive continuous variables at V0. (**b**) Descriptive continuous variables at V1. (**c**) Comorbidities.

**(a)**									
	**Non-Responders**			**Responders**			**Total**		
	***N***	**Mean**	**SD**	***N***	**Mean**	**SD**	***N***	**Mean**	**SD**
Sociodemographic and clinical information									
Age	83	49.47	12.64	48	54.06	12.03	138	51.22	12.44
Education level	78	14.90	3.20	44	14.14	3.35	122	13.37	2.91
Beginning of psychopharmacotherapy	38	34.29	13.78	27	36.63	15.24	65	35.26	23.27
CGI	82	4.98	.875	47	5.09	0.90	135	5.01	0.86
FAST	67	43.72	12.02	37	42.03	13.99	108	42.93	12.75
MADRS	83	29.13	6.54	48	27.31	6.73	136	28.55	6.57
Neuropsychological raw scores									
CODE	69	57.51	20.32	46	50.89	17.17	119	54.79	19.03
SYMBOL	69	26.84	9.77	46	24.57	7.93	119	25.90	8.99
DIGIT_SPAN	68	23.78	6.42	45	23.04	5.61	117	23.46	6.01
TMTA_RT	71	41.54	23.18	45	42.64	14.35	120	42.07	19.97
TMTB_RT	70	97.37	49.55	44	108.68	65.45	118	101.90	55.44
TMTA_E	70	1.29	8.93	45	0.64	1.97	120	0.37	1.58
TMTB_E	69	2.71	15.28	44	1.25	2.83	118	1.03	3.54
FV_semantic	69	27.86	9.36	46	23.30	9.32	119	25.91	9.46
FV_phonologic	69	22.97	8.03	46	19.39	7.26	119	21.39	7.83
ARITMETIC	66	13.67	4.34	41	13.20	3.49	111	13.36	4.01
SIMILITUDES	67	21.64	6.39	45	20.27	5.87	116	21.01	6.19
D2GZF	61	363.80	98.01	38	320.13	93.15	103	344.52	97.13
Baddeley task	51	103.03	74.67	35	65.75	68.57	88	−0.31	6.05
Immediate recall	69	15.35	1.00	45	15.58	0.81	118	15.44	0.92
Free recall 1	69	8.32	1.94	45	7.82	2.35	118	8.10	2.09
Total recall 1	69	14.59	1.86	45	14.18	2.42	118	14.41	2.08
Free recall 2	69	10.00	2.31	45	9.56	2.18	118	9.79	2.29
Total recall 2	69	15.19	1.57	45	15.11	1.42	118	15.18	1.49
Free recall 3	69	11.30	2.61	45	10.51	2.55	118	10.93	2.60
Total recall 3	69	15.41	1.78	45	15.02	1.96	118	15.25	1.83
Sematic distractors	69	0.06	0.29	44	0.36	2.41	117	0.17	1.49
Neutral distractors	69	0.01	0.12	44	0.36	2.41	117	0.15	1.48
Delayed free recall	69	11.36	2.78	45	11.00	3.01	118	11.16	2.84
Delayed total recall	69	15.62	0.89	45	14.87	2.21	118	15.32	1.57
Intrusions	68	0.32	1.00	45	0.82	2.46	117	0.51	1.72
Draw1	43	55.21	22.51	28	50.71	17.64	72	53.47	20.56
Draw2	41	45.78	18.60	28	38.46	14.42	70	42.81	17.17
Neuropsychological standardized scores expressed in standard deviations									
CODE	69	−0.43	1.08	46	−0.75	0.91	119	−0.57	1.01
SYMBOL	69	−0.32	0.95	46	−0.51	0.76	119	−0.41	0.87
DIGIT_SPAN	68	−0.29	1.02	45	−0.37	0.89	117	−0.34	0.96
TMTA_RT	70	−0.17	1.11	45	0.05	0.75	119	−0.10	1.01
TMTB_RT	69	−0.56	1.46	44	−0.07	2.48	117	−0.39	1.90
FV_semantic	66	−0.65	1.16	46	−1.10	1.02	116	−0.86	1.11
FV_phonologic	67	0.01	1.28	46	−0.57	1.17	117	−0.25	1.25
ARITMETIC	66	−0.29	1.12	41	−0.44	0.84	111	−0.38	1.02
SIMILITUDES	67	0.37	1.26	45	0.10	1.21	116	0.24	1.24
Baddeley task	51	0.90	6.23	35	−2.13	5.58	88	88.13	73.32
Free recall 1	69	−0.66	0.82	45	−0.80	1.03	118	−0.73	0.89
Free recall 2	69	−0.66	0.88	45	−0.77	0.88	118	−0.73	0.90
Free recall 3	69	−0.71	1.09	45	−0.97	1.07	118	−0.84	1.09
Delayed free recall	69	-.83	1.12	45	−0.89	1.31	118	−0.88	1.19
Draw1	44	−1.07	1.44	28	−1.26	1.11	73	−1.14	1.31
Draw2	41	−1.05	1.26	28	−1.42	1.03	70	−1.20	1.17
Neuropsychological standardized scores expressed in percentiles									
D2GZF	61	31.71	29.53	38	20.92	23.19	103	26.66	27.49
**(b)**									
	**Non-Responders**			**Responders**			**Total**		
	***N***	**Mean**	**SD**	***N***	**Mean**	**SD**	***N***	**Mean**	**SD**
Sociodemographic and clinical information									
CGI	79	4.51	1.16	48	2.15	1.17	130	3.58	1.64
MADRS	83	24.86	7.86	48	6.52	4.37	133	17.96	11.17
Neuropsychological raw scores									
CODE	45	57.33	16.81	38	56.24	15.34	85	56.75	15.88
SYMBOL	45	29.18	10.83	38	27.61	9.19	85	28.40	9.97
DIGIT_SPAN	42	24.00	6.58	33	24.42	4.55	77	24.10	5.68
TMTA_RT	46	39.52	23.43	36	39.08	13.70	84	39.17	19.45
TMTB_RT	45	91.27	53.40	34	90.47	39.22	81	90.74	47.02
TMTA_E	47	0.36	1.44	36	0.48	1.75	85	0.20	0.48
TMTB_E	47	0.58	2.31	34	0.89	1.54	83	0.59	1.47
FV_semantic	44	24.32	11.92	34	22.59	7.57	79	23.61	10.17
FV_phonologic	44	22.95	11.97	34	19.53	4.53	79	21.37	9.55
ARITMETIC	39	14.23	4.25	32	15.13	3.77	75	22.95	6.46
SIMILITUDES	40	22.78	7.08	33	23.18	5.79	75	22.95	6.46
D2GZF	39	389.64	102.13	32	385.34	68.69	73	385.78	87.60
Baddeley task	30	90.70	22.73	30	85.49	20.73	61	−0.32	1.71
Immediate recall	46	15.57	0.75	36	15.42	0.84	84	15.51	0.78
Free recall 1	46	8.83	2.52	36	8.94	2.55	84	8.89	2.49
Total recall 1	46	14.98	1.25	36	14.58	1.71	84	14.81	1.47
Free recall 2	46	10.15	2.91	36	10.06	2.66	84	10.14	2.76
Total recall 2	46	15.54	0.84	36	15.19	1.56	84	15.39	1.20
Free recall 3	46	11.54	2.87	36	11.22	3.08	84	11.44	2.93
Total recall 3	46	15.76	.74	36	15.61	0.90	84	15.70	0.80
Sematic distractors	46	0.0	0.0	36	0.03	0.16	84	0.01	0.11
Neutral distractors	46	0.0	0.0	36	0.03	0.16	84	0.01	0.11
Delayed free recall	45	11.51	2.88	36	11.72	2.93	83	11.64	2.86
Delayed total recall	45	15.49	1.34	36	15.69	0.90	83	15.58	1.15
Intrusions	44	0.20	0.59	36	0.36	1.15	82	0.29	0.88
Draw1	20	62.50	23.18	21	64.14	18.41	42	63.26	20.38
Draw2	20	49.05	18.21	21	52.81	15.55	42	51.10	16.61
Neuropsychological standardized scores expressed in standard deviations									
CODE	45	−0.49	0.98	38	−0.38	0.92	85	−0.45	0.94
SYMBOL	45	−0.03	1.14	38	−0.12	0.92	85	−0.09	1.03
DIGIT_SPAN	42	−0.31	0.99	33	−0.12	0.73	77	−0.24	0.88
TMTA_RT	46	0.093	0.92	36	0.09	0.75	84	0.10	0.84
TMTB_RT	46	−0.23	1.40	34	−0.06	1.18	82	−0.16	1.30
FV_semantic	51	−0.49	1.33	9	−0.42	1.33	60	0.48	1.28
FV_phonologic	31	0.58	1.95	28	−0.59	0.76	60	0.02	1.60
ARITMETIC	39	−0.12	1.11	32	0.13	1.08	75	0.70	1.34
SIMILITUDES	40	0.68	1.40	33	0.75	1.27	75	0.70	1.34
Baddeley task	30	−0.12	1.84	30	−0.52	1.61	61	88.14	21.55
Free recall 1	46	−0.38	1.00	36	−0.29	1.14	84	−0.33	1.05
Free recall 2	46	−0.55	1.19	36	−0.56	1.10	84	−0.55	1.13
Free recall 3	46	−0.55	1.19	36	−0.66	1.30	84	−0.58	1.22
Delayed free recall	45	−0.71	1.18	36	−0.57	1.35	83	−0.64	1.24
Draw1	21	−0.56	1.58	21	−0.43	1.26	43	−0.50	1.39
Draw2	20	−0.68	1.31	21	−0.41	1.18	42	−0.54	1.22
Neuropsychological standardized scores expressed in percentiles									
D2GZF	39	39.31	30.53	32	37.22	26.40	73	37.56	28.58
**(c)**									
	**Non-Responders**			**Responders**			**Total**		
	***N***	**Percentage**		***N***	**Percentage**		***N***	**Percentage**	
Suicidality	Yes 68 No 9	Yes 88.3 No 11.7		Yes 34 No 11	Yes 75.6 No 24.4		Yes 102 No 20	Yes 83.5 No 16.5	
Panic disorder	Yes 15 No 56	Yes 21.1 No 78.9		Yes 6 No 38	Yes 13.6 No 86.4		Yes 21 No 94	Yes 18.3 No 81.7	
Agoraphobia	Yes 15 No 60	Yes 20 No 80		Yes 7 No 34	Yes 17.1 No 82.9		Yes 22 No 94	Yes 19 No 81	
Social phobia	Yes 16 No 10	Yes 61.5 No 38.5		Yes 11 No 1	Yes 91.7 No 8.3		Yes 27 No 11	Yes 32.5 No 67.5	
PTSD	Yes 3 No 74	Yes 3.9 No 96.1		Yes 4 No 41	Yes 8.9 No 91.1		Yes 7 No 115	Yes 5.5 No 94.5	
Alcohol addiction	Yes 2 No 72	Yes 2.4 No 86.7		Yes 0 No 45	Yes 0 No 100		Yes 2 No 117	Yes 0.8 No 99.2	
GAD	Yes 26 No 51	Yes 33.8 No 66.2		Yes 10 No 34	Yes 22.7 No 77.3		Yes 36 No 85	Yes 30.2 No 69.8	
Antisocial personality disorder	Yes 1 No 76	Yes 1.3 No 98.7		Yes 0 No 44	Yes 0 No 100		Yes 1 No 120	Yes 0.8 No 99.2	

CGI: therapeutic score index; RS: raw score; RT: Reaction Time; E: number of errors.

**Table 2:** Descriptive nominal variables.

	Non-Responders		Responders	
	V0	V1	V0	V1
Sex, male/female	36/47	36/47	18/30	18/30
SSRI, *n*	21	22	19	19
SNRI, *n*	29	29	24	24
Other antidepressants, *n*	44	44	25	25
First generation antipsychotics, *n*	12	12	7	7
Second generation antipsychotics, *n*	34	33	13	13
Mood stabilizers, *n*	22	23	13	13
Anxiolytics, *n*	51	51	24	24
Antiepileptics, *n*	29	29	10	10

SSRI: selective serotonin reuptake inhibitors; SNRI: serotonin and norepinephrine reuptake inhibitors.

**Table 3:** Comparison to the norm 0.

	V0				V1			
	*t*	ddl	Sig. Two Sided	Cohen’s d	*t*	ddl	Sig. Two Sided	Cohen’s d
Non-responders								
CODE	**−3.26**	**68**	**<0.01**	**0.40**	**−3.36**	**44**	**<0.01**	**0.5**
SYMBOL	**−2.82**	**68**	**<0.01**	**0.34**	−0.17	44	0.86	0.03
DIGIT_SPAN	**−2.37**	**67**	**0.02**	**0.28**	**−2.03**	**41**	**<0.01**	**0.31**
TMTA_RT	−1.31	69	0.19	0.15	0.69	45	0.50	0.10
TMTB_RT	**−3.17**	**68**	**<0.01**	**0.38**	−1.12	45	0.27	0.16
FV_semantic	**−4.54**	**65**	**<0.01**	**0.56**	**−2.71**	**50**	**<0.01**	**0.37**
FV_phonologic	0.02	66	0.99	<0.01	1.64	30	0.11	0.30
ARITMETIC	**−2.10**	**65**	**0.04**	**0.26**	−0.65	38	0.05	0.11
SIMILITUDES	**2.38**	**66**	**0.02**	**0.29**	**3.08**	**39**	**<0.01**	**0.49**
Baddeley task	**1.03**	**50**	**0.03**	**0.44**	−0.37	29	0.071	0.14
Free recall 1	**−6.79**	**68**	**<0.01**	**0.80**	**−2.58**	**45**	**0.013**	**0.38**
Free recall 2	**−6.22**	**68**	**<0.01**	**0.75**	**−3.14**	**45**	**<0.01**	**0.46**
Free recall 3	**−5.43**	**68**	**<0.01**	**0.65**	**−3.15**	**45**	**<0.01**	**0.46**
Delayed free recall	**−6.11**	**68**	**<0.01**	**0.74**	**−4.03**	**44**	**<0.01**	**0.60**
Draw1	**−4.90**	**43**	**<0.01**	**0.74**	−1.61	20	0.12	0.35
Draw2	**−5.31**	**40**	**<0.01**	**0.83**	**−2.34**	**19**	**0.03**	**0.52**
Responders								
CODE	**−5.58**	**45**	**<0.01**	**0.82**	**−2.53**	**37**	**0.02**	**0.41**
SYMBOL	**−4.52**	**45**	**<0.01**	**0.67**	−0.83	37	0.41	0.13
DIGIT_SPAN	**−2.75**	**44**	**<0.01**	**0.42**	−0.95	32	0.35	0.16
TMTA_RT	0.49	44	0.63	0.07	0.71	35	0.48	0.12
TMTB_RT	−0.19	43	0.85	0.03	−0.31	33	0.76	0.05
FV_semantic	**−7.31**	**45**	**<0.01**	**1.12**	**−2.71**	**50**	**<0.01**	**0.32**
FV_phonologic	**−3.27**	**45**	**<0.01**	**0.49**	**−4.09**	**27**	**<0.01**	**0.78**
ARITMETIC	**−3.36**	**40**	**<0.01**	**0.52**	0.68	31	0.50	0.12
SIMILITUDES	0.53	44	0.59	0.08	**3.39**	**32**	**<0.01**	**0.59**
Baddeley task	**−2.26**	**34**	**0.03**	**0.38**	−1.77	29	0.09	0.32
Free recall 1	**−5.18**	**44**	**<0.01**	**0.78**	−1.50	35	0.14	0.25
Free recall 2	**−5.92**	**44**	**<0.01**	**0.87**	**−3.07**	**35**	**<0.01**	**0.51**
Free recall 3	**−6.04**	**44**	**<0.01**	**0.91**	**−3.03**	**35**	**<0.01**	**0.51**
Delayed free recall	**−4.58**	**44**	**<0.01**	**0.68**	**−2.54**	**35**	**0.02**	**0.42**
Draw1	**−5.98**	**27**	**<0.01**	**1.14**	−1.56	20	0.13	0.34
Draw2	**−7.28**	**27**	**<0.01**	**1.38**	−1.60	20	0.13	0.35

RS: raw score; SD: standard deviations from the normative mean; RT: Reaction Time; P: percentile.

**Table 4:** Evolution of the scores between V0 and V1.

	Mean	SD	Cohen’s d	*t*	ddl	Sig. Two Sided
Non-responders						
Clinical information						
MADRS	**4.28**	**7.21**	**0.59**	**5.40**	**82**	**<0.01**
Neuropsychological standardized scores expressed in standard deviations						
CODE	−0.03	0.84	0.04	−0.25	41	0.80
SYMBOL	**−0.46**	**0.93**	**0.49**	**−3.22**	**41**	**<0.01**
DIGIT_SPAN	−0.01	0.55	0.02	−0.19	40	0.85
TMTA_RT	−0.22	0.91	0.24	−1.65	44	0.11
TMTB_RT	**−0.43**	**1.34**	**0.32**	**−2.14**	**43**	**0.04**
FV_semantic	−0.03	1.71	0.02	−0.12	44	0.90
FV_phonologic	−0.42	1.54	0.02	−1.50	29	0.14
ARITMETIC	−0.58	2.92	0.20	−1.22	37	0.30
SIMILITUDES	−0.24	0.93	0.26	−1.56	37	0.13
Baddeley task	12.47	64.60	0.19	1.00	26	0.32
Free recall 1	**−0.39**	**1.03**	**0.38**	**−2.61**	**45**	**0.01**
Free recall 2	−0.21	1.27	0.17	−1.15	45	0.26
Free recall 3	−0.22	1.11	0.20	−1.33	45	0.19
Delayed free recall	−0.19	1.07	0.18	−1.22	44	0.23
Draw1	**−0.71**	**1.52**	**0.47**	**−2.11**	**19**	**0.05**
Draw2	−0.28	1.30	0.22	−0.90	17	0.38
Neuropsychological standardized scores expressed in percentiles						
D2GZF	−5.16	28.09	0.18	−1.09	34	0.28
Responders						
Clinical information						
MADRS	**20.79**	**6.71**	**3.10**	**21.46**	**47**	**<0.01**
Neuropsychological standardized scores expressed in standard deviations						
CODE	**−0.38**	**0.83**	**0.46**	**−2.80**	**37**	**<0.01**
SYMBOL	**−0.45**	**1.08**	**0.42**	**−2.54**	**37**	**0.01**
DIGIT_SPAN	**−0.30**	**0.83**	**0.36**	**−2.05**	**32**	**0.05**
TMTA_RT	−0.11	0.90	0.12	−0.72	34	0.48
TMTB_RT	−0.31	1.33	0.23	−1.36	32	0.18
FV_phonologic	−0.18	1.31	0.14	−0.72	27	0.48
ARITMETIC	**−1.43**	**2.81**	**0.51**	**−2.80**	**29**	**<0.01**
SIMILITUDES	**−0.84**	**1.02**	**0.82**	**−4.72**	**32**	**<0.01**
Baddeley task	−22.98	77.37	0.30	−1.54	26	0.13
Free recall 1	**−0.57**	**1.06**	**0.54**	**−3.22**	**35**	**<0.01**
Free recall 2	−0.29	1.11	0.26	−1.59	35	0.12
Free recall 3	**−0.38**	**1.13**	**0.34**	**−2.05**	**35**	**0.05**
Delayed free recall	−0.38	1.40	0.27	−1.62	35	0.11
Draw1	−0.67	1.64	0.41	−1.81	19	0.08
Draw2	**−0.98**	**1.46**	**0.67**	**−3.01**	**19**	**<0.01**
Neuropsychological standardized scores expressed in percentiles						
D2GZF	**−15.14**	**19.00**	**0.80**	**−4.14**	**26**	**<0.01**

RS: raw score; SD: standard deviations from the normative mean; RT: Reaction Time; P: percentile.

**Table 5:** Comparison of responders and non-responders at V1.

	*t*	ddl	*p* Value	Mean Difference	Cohen’s d
Clinical information					
MADRS	1.52	129	0.13	1.82	0.27
ΔMADRS	**12.94**	**129**	**<0.01**	**16.51**	**2.35**
Neuropsychological standardized scores expressed in standard deviations					
CODE	1.65	113	0.10	0.32	0.31
SYMBOL	1.10	113	0.27	0.18	0.21
DIGIT_SPAN	0.38	111	0.70	0.07	0.07
TMTA_RT	−1.21	113	0.23	−0.23	0.23
TMTB_RT	−1.32	111	0.19	−0.49	0.25
FV_semantic	**2.15**	**110**	**0.03**	**0.46**	**0.41**
FV_phonologic	**2.39**	**111**	**0.02**	**0.57**	**0.46**
ARITMETIC	0.72	105	0.47	0.44	0.14
SIMILITUDES	1.13	110	0.26	0.27	0.22
Baddeley task	**2.35**	**84**	**0.02**	**37.28**	**0.51**
Free recall 1	0.76	112	0.45	0.13	0.15
Free recall 2	0.66	112	0.51	0.11	0.13
Free recall 3	1.21	112	0.23	0.25	0.23
Delayed free recall	0.30	112	0.77	0.07	0.32
Draw1	0.59	70	0.56	0.19	0.33
Draw2	1.27	67	0.21	0.37	0.32
ΔCODE	−1.85	78	0.07	−0.34	0.42
ΔSYMBOL	0.05	78	0.96	0.01	0.01
ΔDIGIT_SPAN	−1.75	72	0.08	−0.28	0.42
ΔTMTA_RT	0.57	78	0.57	0.12	0.13
ΔTMTB_RT	0.38	75	0.70	0.11	0.09
ΔFV_semantic	0.64	56	0.52	0.24	
ΔFV_phonologic	−1.22	66	0.23	−0.85	0.17
ΔARITMETIC	**−2.60**	**69**	**0.01**	**−0.60**	**0.30**
Baddeley task	−1.83	52	0.07	−35.44	0.50
ΔFree recall 1	−0.76	80	0.50	−0.18	0.17
ΔFree recall 2	−0.29	80	0.77	−0.08	0.06
ΔFree recall 3	−0.67	80	0.50	−0.17	0.15
ΔDelayed free recall	−0.68	79	0.50	−0.19	0.15
ΔDraw1	0.01	38	0.92	0.05	0.03
ΔDraw2	−1.57	36	0.13	−0.71	0.51
Neuropsychological standardized scores expressed in percentiles					
D2GZF	1.91	97	0.60	10.79	0.39
ΔD2	−1.59	60	0.12	−9.97	0.41

RT: Reaction Time; Δ: V1 − V0.

## Data Availability

Data are available on reasonable request from the first author.
